# Development of CpG-Oligodeoxynucleotides for Effective Activation of Rabbit TLR9 Mediated Immune Responses

**DOI:** 10.1371/journal.pone.0108808

**Published:** 2014-09-30

**Authors:** Tsung-Hsien Chuang, Chao-Yang Lai, Ping-Hui Tseng, Chiun-Jye Yuan, Li-Chung Hsu

**Affiliations:** 1 Immunology Research Center, National Health Research Institutes, Miaoli, Taiwan; 2 Program in Environmental and Occupational Medicine, Kaohsiung Medical University, Kaohsiung, Taiwan; 3 Institute of Biochemistry and Molecular Biology, National Yang-Ming University, Taipei, Taiwan; 4 Department of Biological Science and Technology, National Chiao Tung University, Hsinchu, Taiwan; 5 Institute of Molecular Medicine, National Taiwan University, Taipei, Taiwan; Whitehead Institute, United States of America

## Abstract

CpG-oligodeoxynucleotides (CpG-ODN) are potent immune stimuli being developed for use as adjuvants in different species. Toll-like receptor 9 (TLR9) is the cellular receptor for CpG-ODN in mammalian cells. The CpG-ODN with 18–24 deoxynucleotides that are in current use for human and mouse cells, however, have low activity with rabbit TLR9. Using a cell-based activation assay, we developed a type of CpG-ODN containing a GACGTT or AACGTT motif in 12 phosphorothioate-modified deoxynucleotides with potent stimulatory activity for rabbit TLR9. The developed CpG-ODN have higher activities than other developed CpG-ODN in eliciting antigen-nonspecific immune responses in rabbit splenocytes. When mixed with an NJ85 peptide derived from rabbit hemorrhagic disease virus, they had potent activities to boost an antigen-specific T cell activation and antibody production in rabbits. Compared to Freund’s adjuvant, the developed CpG-ODN are capable of boosting a potent and less toxic antibody response. The results of this study suggest that both the choice of CpG-motif and its length are important factors for CpG-ODN to effectively activate rabbit TLR9 mediated immune responses.

## Introduction

Toll-like receptors (TLRs) are a family of pattern recognition receptors. These receptors are essential for innate immune cells to detect a wide variety of pathogen-associated molecular patterns – such as those of lipids, lipoproteins, glycans, proteins, and nucleic acids – to elicit host immune responses. Ten TLRs have been identified in human cells, and thirteen identified in murine cells. TLR9 detects bacterial DNA, and belongs to a subfamily of intracellular TLRs that contains TLR3, TLR7, TLR8, and TLR9 [1]–[6]. Synthetic phosphorothioate-modified CpG-oligodeoxynucleotides (CpG-ODN) mimic the immunostimulatory activity of bacterial DNA [7]–[12]. Activation of TLR9 by CpG-ODN triggers sequential recruitment of signaling proteins – including MyD88, IRAK, and TRAF6 – to form a complex that in turn activates downstream TAK, leading to activation of important transcription factors, including NF-κB and interferon regulatory factors [13]–[15]. Activation of TLR9 can result in a number of immunological effects, including increasing production of T helper (Th)1 polarized cytokines, up-regulation of major histocompatibility complex (MHC), co-stimulatory molecules, and activation and enhancement of B cell proliferation to increase antibody production. Because of these potent immunostimulatory effects, CpG-ODN are being investigated for a broad range of therapeutic applications in humans – including immunotherapies for allergies, cancer, and infectious diseases – and being utilized as adjuvants in different species [16]–[24].

In general, a CpG-ODN contains 18–24 phosphorothioate-modified deoxynucleotides, with one or more copies of CpG-deoxynucleotides containing hexamer motifs (CpG-motifs); and its immunostimulatory activity is dependent on the number of CpG-motifs, as well as on the position, spacing, and surrounding bases of the CpG-motifs. The immunostimulatory activity of a CpG-ODN can differ across species. Current knowledge is that this species-specific property is determined by the nucleotide context of the CpG-motifs within the CpG-ODN. For example, CpG-ODN containing the GTCGTT motif generate higher immune responses in humans and various domestic animals than those with the GACGTT motif; in contrast, the latter are more potent in activation of murine cells [9]–[12]. The species-specific activity of a CpG-ODN is due to a different degree of TLR9 activation in different species [25], [26].

Rabbits are a popular pet, trailing only dogs and cats. In addition rabbits are commonly used in laboratories for production of antibodies. Although CpG-ODN have been investigated as immunological adjuvant in a wide variety of animals and fish [22]–[24], previous studies have shown that CpG-ODN with 18–24 deoxynucleotides in current use for the activation of human or mouse cells have low activity to rabbit TLR9 (rabTLR9) [27], [28]. Here, we report development of a type of CpG-ODN which robustly activates to rabTLR9 and effectively induces antigen-nonspecific and -specific immune responses in rabbits. Moreover, they are potent and less toxic as an adjuvant to boost antibody production in rabbits. Distinct from those CpG-ODN in current use for humans and mice, these new CpG-ODN have a short length. They contain a GACGTT or AACGTT motif in 12 phosphorothioate-modified deoxynucleotides.

## Materials and Methods

### Ethics statement

The animal experiments were approved by the Institutional Animal Care and Use Committee (IACUC) of the National Health Research Institutes, Taiwan. New Zealand white rabbits and C57/B6J mice were maintained and handled in accordance with the guidelines.

### Reagents and antibodies

CpG-ODN were purchased from Invitrogen or Genomics Biosci and Tech. Ovalbumin and aluminum hydroxide gel were purchased from Invivogen. Freund’s complete adjuvant and incomplete adjuvant were purchased from Thermo Scientific. Luciferase assay reagents were purchased from Promega.

### TLR9 activation assays

Rabbit, human, and mouse TLR9 expression constructs were generated as previously reported [27]. To perform TLR9 activation assays, HEK293 cells were grown in Dulbecco’s minimum essential medium (DMEM) supplemented with 10% fetal bovine serum, plated on 24-well plates, and allowed to adhere overnight. These cells were co-transfected using PolyJet (SignaGen) with TLR9 expression vector, β-galactosidase plasmid, and an NF-κB driven luciferase reporter plasmid; and treated with 2µM or with different concentrations of various CpG-ODN, as indicated, on the next day for 7h. The cells were lysed; and luciferase activity in each sample was determined. Relative luciferase activities were calculated as fold induction compared to an unstimulated control. The data are expressed as the means ± SD (n = 3 independent experiments).

### RT-PCR analysis of cytokine inductions

Splenocytes were prepared from mice and rabbits, and were maintained in RPMI medium supplemented with 10% fetal bovine serum. For analysis of cytokine inductions, cells were treated with 2µM of CpG-ODN for 4h. Total RNAs were isolated with an RNeasy mini kit (Qiagen) following the manufacturer’s protocol. First-strand cDNA libraries were then synthesized from the collected total RNA samples using a SuperScript preamplification kit (Invitrogen); and PCR amplifications were performed using an Expand PCR kit (Roche). The sequence of the PCR primers for rabbit GAPDH, IL-6, IL-8, and IFN-α are 5′-ccgagtacgtggtggaatccactg-3′ and 5′-ctgtagccaaattcgttgtcatacc-3′, 5′- gtcccctcggctgctcgctgg -3′ and 5′-caggctgaccgcaacggctggc-3′, 5′-gacacggattggtacagagcttcg -3′ and 5′- cttggaactcatggcctgaccaacag -3′, and 5′-ctccaagtccctctgctctctgg-3′ and 5′-aggcacaagggctgtattgcttc-3′, respectively. PCR products were visualized by electrophoresis on a 1% agarose gel after staining with ethidium bromide.

### Cell proliferation assays

Proliferation of splenocytes was measured by CellTiter 96 AQ_ueous_ Non-Radioactive Cell Proliferation (MTS) assay according to the manufacturer’s instructions (Promega). Briefly, rabbit and mouse spleen cells (1×10^5^/well) were treated with 2µM of CpG-ODN for 48h. MTS/PMS solutions were added into each well. After 2h the absorbencies at 490nm were measured by an Envision Alpha Multilabel Reader (PerkinElmer).

### ELISA assay for cytokine and total IgM production

Rabbit and mouse splenocytes (1×10^6^/ml) were treated with 2µM of CpG-ODN for 2 days. The cell culture media were collected for measurement of cytokine and IgM inductions. The total rabbit IgM was measured with a Rabbit IgM ELISA Kit (GenWay Biotech), and mouse cytokines and IgM were measured with ELISA kits from eBiosciences, according to the manufacturers’ instructions.

### Rabbit Immunization

RHDV NJ85 peptide (GSASYSGNNATNVLQFWYA) [31] was synthesized and conjugated onto keyhole limpet hemocyanin (KLH). This peptide was dissolved in DMSO and then diluted to immunization concentration with PBS. Each rabbit was immunized with NJ85 peptide or ovabumin, with or without different CpG-ODN or CFA/IFA, as indicated.

### Isolation of rabbit peripheral CD8 T cells

For isolation of rabbit peripheral CD8 T cells, 10ml of rabbit whole blood were collected and centrifuged for buffy coat collection. Rabbit red blood cells were then lysed by BD Pharm Lyse lysing solution (BD Bioscience). The buffy coat cells were incubated with mouse anti-rabbit CD8 IgG (AbD Serotec), followed by incubation with anti-Mouse IgG MicroBeads (Miltenyi Biotec). The rabbit CD8 T cells were then purified using a magnetically activated cell sorting (MACS) system (Miltenyi Biotec) according to the manufacturer’s instructions.

### Measurement of rabbit IFN-γ

For measurement of IFN-γ productions from NJ85 peptide specific CD8 T cells, the isolated CD8 T cells were cultured in RPMI1640 with 10% FBS and treated with 10µg/ml of NJ85 peptide for 24h. Supernatants were then collected and measured for IFN-γ by using ELISA Kit (USCN Life Science Inc.).

### Measurement of anti-NJ85 and anti-OVA antibodies

NJ85 peptide and ovabumin were dissolved in ELISA Coating Buffer (eBioscience) and coated (2µg/well) onto a 96-well ultra-clear polypstyrene microtiter plate overnight at 4°C. The plate was washed with wash solution (1X PBS and 0.05% Tween 20) and blocked with 1x assay diluent (eBioscience) for 1h at room temperature. After four washes, 100µl of diluted serum was added and incubated overnight at 4°C. Subsequently, after five washings, 100µl of 1/5000 diluted biotin-conjugated anti-rabbit IgG (KPL Inc.) was added, and the mixtures were incubated for 1h. After washes, 100µl/well of 1/500 diluted Avidin-HRP (eBioscience) were added and incubated for 30min. After four washes, 100µl of TMB (eBioscience) was added to each well for colar development. The reaction was stopped by adding of 50µl of 2N H_2_SO_4_. The absorbance was determined at 450nm using an Envision Alpha Multilabel Reader (PerkinElmer).

### Histology analysis

For histology analysis, the isolated tissues were immersed in 10% formalin. These samples were then embedded in paraffin wax and sections were hematoxylin-eosin (HE) stained.

### Statistical analysis

Groups of data are expressed as mean ± SD. Statistical analyses were performed using Student’s t-test. All groups were from three or more independent experiments. P<0.05 was considered statistically significant.

## Results

### Development of CpG-ODN for effective activation of rabTLR9

**Figure 1:**
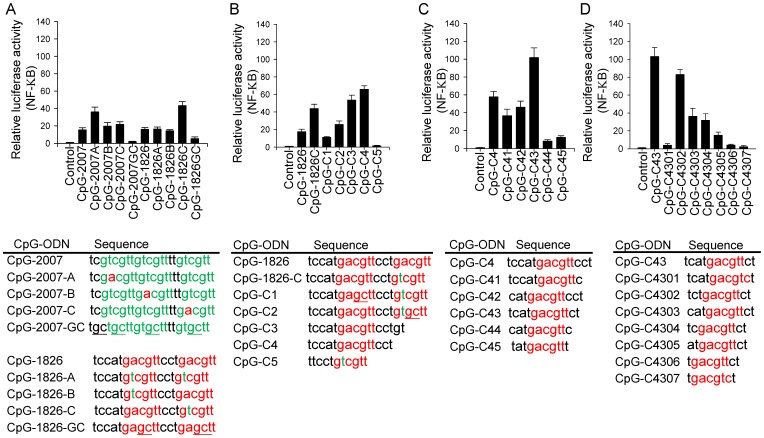
Development of CpG-ODN for activation of rabTLR9. Activation of rabTLR9 by (A) CpG-ODN derived from CpG-2007 and CpG-1826, (B) CpG-ODN derived from CpG-1826C, (C) CpG-ODN derived from CpG-C4, and (D) CpG-ODN derived from CpG-C43. HEK 293 cells were co-transfected with expression vector for rabTLR9 and an NF-κB drivened luciferase-reporter gene, and treated with 2µM of different CpG-ODN as indicated for 7h. Relative luciferase activities were then determined. Data shown represent mean ± SD (n = 3 independent experiments). The sequences of CpG-ODN used in this study are shown at the bottom of the plots.

Previous studies showed that in contrast to human (h)TLR9 and mouse (m)TLR9, rabbit (rab)TLR9 has a broad ligand recognition profile to recognize CpG-ODN in current use for humans and mice with either GTCGTT motifs or GACGTT motifs [27], [28]. We were curious to learn how a CpG-ODN would act on rabTLR9 if the CpG-ODN contained both the GTCGTT and GACGTT motifs. CpG-2007 and CpG-1826, which were optimized for human and mouse cells, contain 3 copies of GTCGTT motifs and 2 copies of GACGTT motifs, respectively. They were modified into different CpG-ODN containing both types of CpG-motifs. The activities of these CpG-ODN were investigated with a cell-based assay in which the activation of rabTLR9 was measured by luciferase reporter activity. Interestingly, we found that a CpG-1826-C with a copy of the GACGTT motif followed by a copy of the GTCGTT motif had stronger activity than its parental CpG-2007, CpG-1826, and other modified CpG-ODN tested (Figure 1A).

In an attempt to develop optimized CpG-ODN for effective activation of rabTLR9 and immune responses in rabbits, we asked if a copy of GACGTT and a copy of the GTCGTT motif in a CpG-ODN are indeed required for strong activity. The CpG-1826-C was further modified into several different CpG-ODN, with the CpG-deoxynucleotides reversed in the two different types of motifs, or with the N-terminal or C-terminal GACGTT or GTCGTT motif deleted. Of these CpG-ODN, a CpG-C4 generated the best activity with rabTLR9. This CpG-ODN contains 14 phosphorothioate-modified deoxynucleotides and is a truncated form of the CpG-1826-C with the C-terminal GTCGTT motif deleted. This suggested that the C-terminal GTCGTT motif is not required for strong activation of rabTLR9 (Figure 1B). Moreover, the increased activity from CpG-1826C, CpG-C3, and CpG-C4 – which contain reduced lengths of 20, 16, and 14 phosphorothioate-modified deoxynucleotides, respectively – led us to speculate that the length of a CpG-ODN may be critical for strong activation of rabTLR9.

### Activation of rabTLR9 by CpG-C46 and CpG-C4609

**Figure 2:**
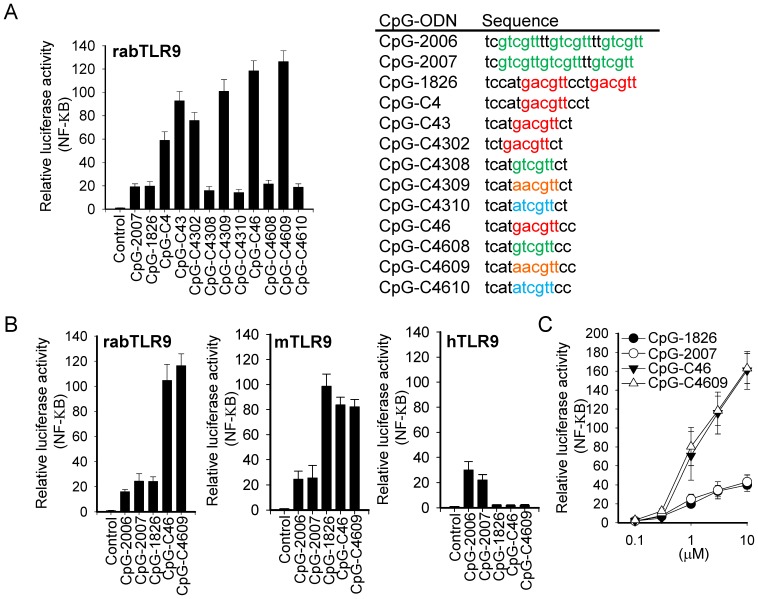
Activation of TLR9s by CpG-ODN developed for rabbits. HEK 293 cells were transfected with expression vector for (A, C) rabTLR9 and for (B) rabbit, mouse, and human TLR9, as indicated, along with an NF-κB controlled luciferase-reporter gene, and treated with 2µM of CpG-ODN (A, B) or with different concentrations of CpG-ODN (C) for 7h. Relative luciferase activities were then determined. Data shown represent mean ± SD (n = 3 independent experiments). The sequences of CpG-ODN used in this study are shown on the right of panel A.

We further reduced the length and changed the 5 and 3 phosphorothioate-modified deoxynucleotides to modify the CpG-ODN. Results from cell-based activation assay indicated that CpG-ODN containing 12 phosphorothioate-modified deoxynucleotides with a GACGTT motif or AACGTT motif (such as CpG-C43, -C4309, -C46, and -C4609) have strong activities with rabTLR9, whereas those with the GTCGTT motif or ATCGTT motif (such as CpG-C4308, -C4310, -C4608, and -C4609) have weak activities (Figure 1C and Figure 2A). CpG-ODN containing 11 phosphorothioate-modified deoxynucleotides, such as CpG-C4302, had good activity with rabTLR9, although not as good as that of CpG-C43; but further trimming of the length reduced the activity dramatically (Figure 1D).

The CpG-C46 and CpG-C4609, which contain GACGTT and AACGTT motifs, respectively, in 12 phosphorothioate-modified deoxynucleotides, were selected for further study to compare their activities with CpG-2006, -2007 (optimized for human cells), and −1826 (optimized for mouse cells) for rabTLR9, mTLR9, and hTLR9 activation in cell-based activation assays. Compared to these CpG-ODN, CpG-C46, and CpG-C4609 were much more potent in activation of rabTLR9 at 2µM concentration and under different concentrations (Figure 2, B and C), and they showed better activity than CpG-2006 and CpG-2007 in activating mTLR9 but no activity with hTLR9 (Figure 2B).

### Activation of antigen-nonspecific and -specific immune responses in rabbit by CpG-4609

**Figure 3:**
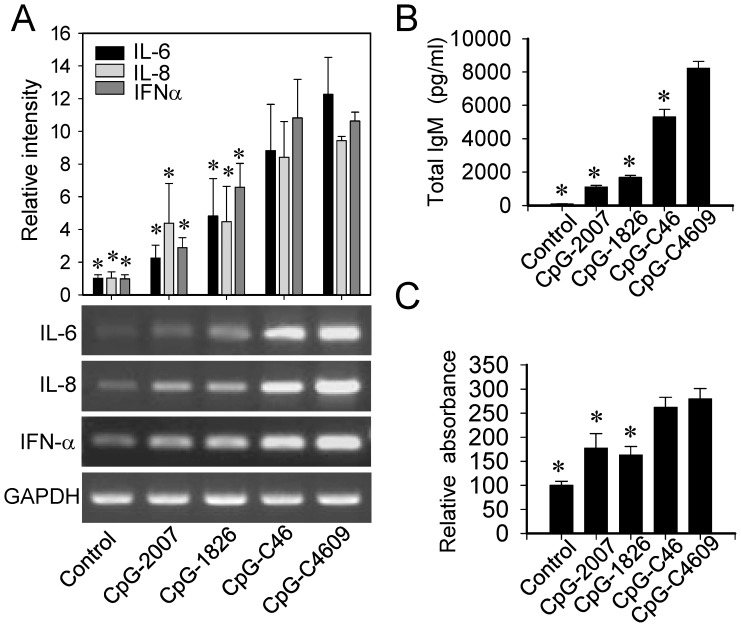
Activation of antigen-nonspecific immune responses by CpG-ODN developed for rabbits. Rabbit splenocytes were stimulated with 2µM of different CpG-ODN. (A) Induction of cytokines was analyzed by RT-PCR. (B) Production of IgM was determined by ELISA. (C) Proliferation of splenocytes was measured by MTS assay. Data shown represent mean ± SD (n = 3 independent experiments).*P<0.05 *vs.* the induction by CpG-C4609.

The immunostimulatory activities of these CpG-ODN were further investigated with rabbit splenocytes. Cytokine production induced in the cells was analyzed by RT-PCR. Cell proliferation was measured by MTS assay, while IgM production was determined by ELISA. In line with their performances in the cell-based rabTLR9 activation assays, the CpG-C46 and -C4609 had higher activities than the CpG-2007 and−1826 in activating IL-6, IL-8, and IFN-α induction (Figure 3A); and IgM production in splenocytes (Figure 3B). CpG-C46 and -C4609 also had higher activities in increasing proliferation of rabbit splenocytes (Figure 3C).

**Figure 4:**
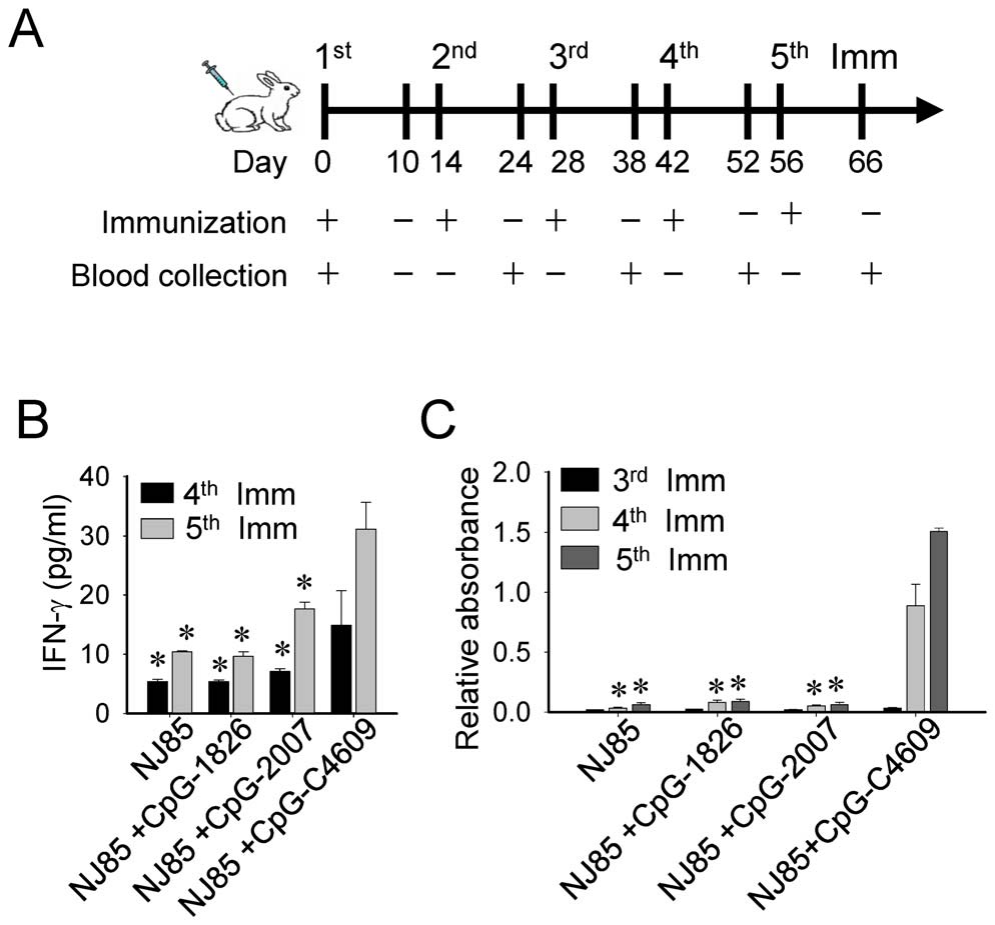
Activation of antigen-specific immune responses by CpG-ODN developed for rabbits. Rabbits were immunized with 100µg of RHDV NJ85 peptide mixed with or without 50µg of CpG-ODN, as indicated. (A) The cycle of immunization and serum collection followed a 14-day-interval schedule, as shown. (B) Inductions of IFN-γ from CD8 T cells by NJ85 were measured by ELISA. (C) Titers of anti-NJ85 antibody in serum samples were measured by ELISA. Data shown represent mean ± SD (n = 3 independent experiments). *P<0.05 *vs.* the induction by CpG-C4609.

Rabbit hemorrhagic disease virus (RHDV) causes hemorrhagic disease in rabbits [29], [30]. An NJ85 peptide derived from one of the regions for RHDV to interact with rabbit tissue cells has been shown to effectively elicit anti-RHDV immune responses and has been suggested as a vaccine candidate for RHDV [31]. We next utilized this NJ85 peptide to investigate adjuvant activities of these CpG-ODN in boosting antigen-specific immune response, including activation of antigen-specific T cells and induction of antigen-specific antibody responses. The NJ85 peptide (100µg) and CpG-ODN (50µg) were mixed and injected subcutaneously into rabbits. Injection and blood samples were collected at 14-day intervals (Figure 4A). Activation of antigen-specific T cells was determined by purification of CD8 T cells and measurement of NJ85-induced IFN-γ production from these cells. Titers of anti-NJ85 antibody generated in serums were measured by ELISA. The results indicated that CpG-C4609 was more potent than CpG-1826 and CpG-2007 in induction of these antigen-specific immune responses in rabbits (Figure 4, B and C), suggesting that CpG-C4609 had a higher adjuvant activity.

### Activation of antigen-nonspecific and -specific immune responses in mouse by CpG-4609

**Figure 5:**
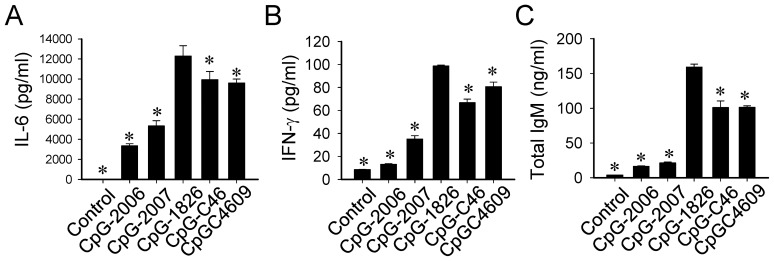
Activation of mouse splenocytes by CpG-ODN developed for rabbits. Mouse splenocytes were stimulated with 2µM of different CpG-ODN: CpG-2006 and CpG-2007, optimized for humans; CpG-1826, optimized for mice; and CpG-C46 and CpG-C4609, developed for rabbits. Induction of (A) IL-6 and (B) IFN-γ, and production of (C) IgM, were determined by ELISA. Data shown represent mean ± SD (n = 3 independent experiments). *P<0.05 *vs.* the induction by CpG-1826.

**Figure 6:**
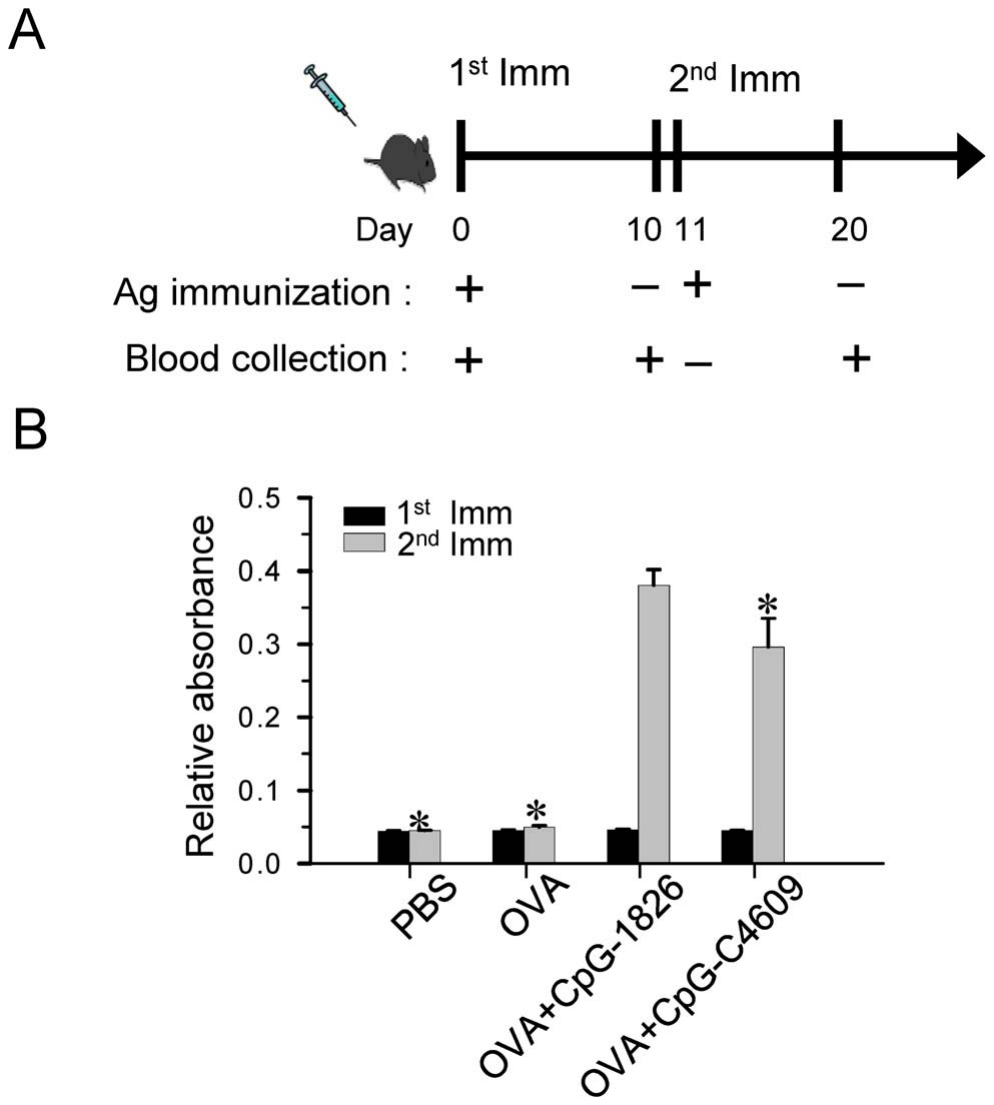
Efficiency of the developed CpG-ODN in boosting antibody production in mice. (A) A 10-day-interval schedule, as shown, was adopted for each cycle of immunization and serum collection from C57/B6J mice. (B) 1µg of OVA was formulated with/without 5µg of CpG-ODN in PBS for each mouse. Anti-OVA antibody titers were measured by ELISA. Data shown represent mean ± SD (n = 3 independent experiments). *P<0.05 *vs.* the induction by CpG-1826.

Since CpG-C46 and -C4609 also had good activities with mTLR9 (Figure 2B **)**, the activities of these CpG-ODN to induce immune responses in mice were investigated. Mouse splenocytes were prepared and stimulated with different CpG-ODN. Similar to their activities in the mTLR9 activation assay (Figure 2B), CpG-C46 and CpG-C4609 had higher activities than the CpG-2006 and -2007 in inducing IL-6 (Figure 5A), IFN-γ (Figure 5B), and IgM (Figure 5C) production in these cells, although the activities were not as strong as with CpG-1826. In line with this, when mice were injected with a mixture of CpG-ODN (5µg) and a conventional protein antigen, ovalbumin (1µg), the CpG-C4609 also activated antigen-specific antibody production (Figure 6).

### CpG-C4609 is a potent and less toxic adjuvant for boosting antibody production in rabbits

**Figure 7:**
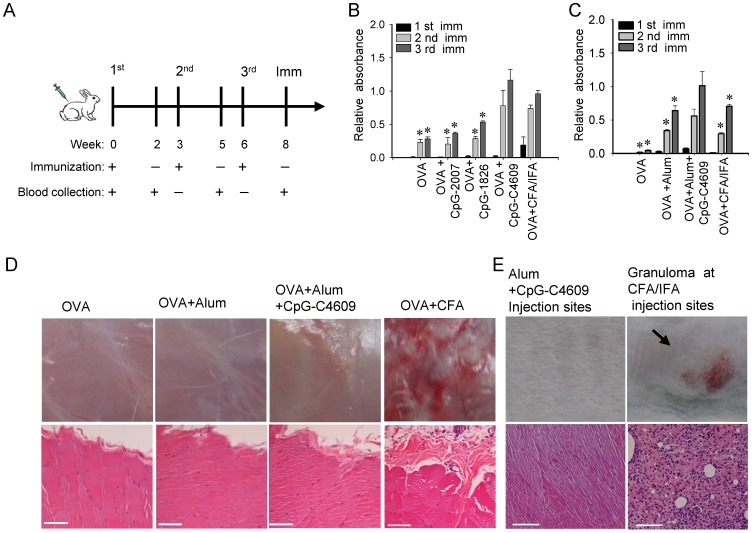
Efficacy and safety of the developed CpG-ODN in boosting antibody production in rabbits. (A) A three-week-interval schedule, as shown, was adopted for each cycle of immunization and serum collection. (B) 10µg of OVA was formulated with 50µg of CpG-ODN in PBS or CFA/IFA. (C) 3µg of OVA was formulated with 50µg of CpG-ODN, 1% of aluminum hydroxide gel (alum), in PBS or CFA/IFA, to determine the efficacy and safety of CpG-C4609 as an adjuvant to boost antibody production. Anti-OVA antibody titers were measured by ELISA. Data shown represent mean ± SD (n = 3 independent experiments). *P<0.05 *vs.* the induction by CpG-C4609 or the induction by alum plus CpG-C4609. (D) Upper panels: subcutaneous tissues at the injection sites were examined on the second day after injection of different combinations of antigens and adjuvants, as indicated. Lower panels: HE staining of sections taken from these tissues. Photos show a set of representative results of three independent experiments.(E) Upper panels: representative photos of granuloma appeared at CFA/IFA injection sites (right), and skin at the alum plus CpG-C4609 injection sites (left). Lower panels: HE staining of sections taken from the granuloma (right), and tissue from the alum plus CpG-C4609 injection sites (left). The right panels show a representative granuloma developed in 30% of CFA/IFA injected rabbits (n = 20). The left panels show injection sites of an alum plus CpG-C4609 injected rabbit which is representative for rabbits (n = 20) injected without adjuvant or with adjuvant other than CFA/IFA.

We further investigated the capability of the developed CpG-ODN as adjuvants to boost antibody production in rabbits, and compared the safety and efficacy of CpG-C4609 with CFA/IFA in boosting antibody production. Ovalbumin was injected subcutaneously into rabbits after being mixed with these adjuvants. A three-week interval, as shown in Figure 7A, was adopted for each cycle of immunization and blood collection. When 10µg of ovalbumin was mixed with 50µg of CpG-C4609 in PBS, the elicited antibody responses were as good as that elicited by CFA/IFA (Figure 7B). When 3µg of ovalbumin was mixed with 50µg of CpG-C4609 in 1% of aluminum hydroxide gel (alum), the induced antibody responses were better than those elicited by alum alone and by CFA/IFA (Figure 7C).

The adverse effects of these adjuvants were further examined. On the second day after sc injection, massive lesions and inflammation appeared at the subcutaneous tissues of the CFA injection sites. In contrast, much less or no tissue damage was seen at the alum or alum-mixed CpG-C4609 injection sites (Figure 7D, upper panels). Similarly, histopathologic analysis with HE staining revealed leukocyte infiltration and damage of subcutaneous tissue at the CFA injection sites but not at the areas injected with alum and alum-mixed CpG-C4609 (Figure 7D, lower panels). Moreover, granuloma, as shown in Fig. 7E, was sometimes seen at the CFA/IFA injection sites; but this was not seen at injection sites of rabbit injected without adjuvant or with adjuvant other than CFA/IFA, such as the alum-mixed CpG-C4609 injection sites. This indicates that CpG-C4609 is less toxic than CFA/IFA and is a safer adjuvant to elicit potent antibody response in rabbits.

## Discussion

In this study we have developed a type of CpG-ODN that effectively targets TLR9 to elicit immune responses in rabbits. These CpG-ODN consist of a GACGTT motif or an AACGTT motif. These two types of CpG-motifs are known to be required for a CpG-ODN to robustly activate mouse cells [9]–[12]. In this regard, it is not surprising that the developed CpG-C4609 also activate antigen-specific and -nonspecific immune responses in mice well, although not as well as the CpG-1826 developed for mice. Of interest is that although mice and rabbits are both rodents, the overall protein similarities between mTLR9 and rabTLR9 are not as great as those between rabTLR9 and TLR9 from other animals [27]. That the same CpG-motif is required for strong activation of the TLR9s of both mouse and rabbit suggests that the CpG-ODN binding sites within these two TLRs could be more conserved than the whole TLR9 proteins from these two species.

The developed CpG-ODN, however, is distinct in its length from the CpG-ODN currently in frequent use for humans and mice. A short length of 12 deoxynucleotides is required for effective activation of rabTLR9. In contrast, longer CpG-ODN contain reduced activities. Both CpG-C46 and CpG-C4609 contain 4 deoxynucleotides before their CpG-motif and 2 deoxynucleotides after the CpG-motif. This is consistent with the critical role of the CpG-motif in activation of TLR9s [9]–[12]. TLR3, TLR7, TLR8, and TLR9 comprise a subfamily of TLRs that localize in intracellular compartments to detect nucleotide-based structures [3]–[5]. The crystal structures of extracellular domains of TLR3 and TLR8 have been determined. The two ligand binding sites of TLR3 are located at the N-terminus and C-terminus of its extracellular domain. Thus, dsRNA must with at least 40 nucleotides (the sequence can be random) to reach these two binding sites for a strong activation of TLR3. In contrast, the binding sites of TLR8 for its small molecular weight ligands are located at LRR11-14 and LRR16-18, which are in the central curve region of its horseshoe-like extracellular domain [32]–[36]. The structure and the ligand binding sites of TLR9 have not yet been determined. Nevertheless, our finding that rabTLR9 can be activated by a short CpG-ODN containing 12 phosphorothioate-modified oligodeoxynucleotides with a sequence-specific CpG-hexamer motif suggests that the binding of CpG-ODN to TLR9 might be more like that of TLR8 and less similar to that of TLR3. Indeed, computational modeling and experimental data have shown the involvement of LRR11 in CpG-ODN binding to TLR9 [37], [38].

CpG-ODN optimized for humans are being investigated for their application in immunotherapies for allergies, cancer, and infectious diseases, and as vaccine adjuvants. CpG-ODN optimized for mice are being used as adjuvants to activate immune responses for generation of monoclonal antibodies. It is known that CpG-ODN with GTCGTT motif for activation of human cells have better activity in domestic animals than CpG-ODN with GACGTT motif for activation of mouse cells [17]–[24]. But so far no CpG-ODN has been optimized for any species other than mouse and human. Moreover, it is known that CpG-ODN are less potent immune activators in humans and domestic animals than in mice, which is attributed to the differences in the cellular expression of TLR9 in these species. In mice, TLR9 is expressed in macrophages and all B cells and dendritic cell populations. In humans, expression of TLR9 is limited to plasmacytoid dendritic cells and some B cell subsets; moreover, the cellular expression profile in large domestic animals could be more like that of humans [24]. Our results suggest that the low activities of CpG-ODN in current use on TLR9s could be another reason for the low activity of CpG-ODN in humans and domestic animals. The results of this study also suggest that it would be possible to target TLR9 from individual species to optimize CpG-ODN for that species, and that both the choice of CpG-motif and its length are important factors in developing CpG-ODN for different species.

Rabbit polyclonal antibodies are commonly generated in laboratories for various studies requiring protein detection. In addition, rabbit hybridoma fusion cell lines and phage display technologies have been developed, making it possible to generate large amounts of rabbit monoclonal antibodies [39]–[41]. Freund’s adjuvant is widely utilized for effectively boosting antibody production in rabbit. However, this adjuvant has the strong adverse effect of causing inflammatory responses and lesions of tissues around the injection site [42], [43]. In addition, rabbits’ popularity as pets (after only dogs and cats) heightens the needs to develop vaccines to protect rabbits against virus infections [29], [30]. The CpG-ODN developed in this study apparently can be an adjuvant for rabbits because of their potency and safety in induction of antigen-specific and -nonspecific immune responses in rabbits. Diverse delivery strategies and routes for administration of CpG-ODN have been investigated in many studies. A CpG-ODN can be conjugated to antigen, carried together with antigen by liposomes or nanoparticles, and administrated by intradermal, intramuscular, and subcutaneous injections, or by nasal spraying [44]–[46]. These further increase the potential of applications with CpG-ODN developed in this study in rabbits.
